# Long-Term Survival in a Large Cohort of Patients with Venous Thrombosis: Incidence and Predictors

**DOI:** 10.1371/journal.pmed.1001155

**Published:** 2012-01-10

**Authors:** Linda E. Flinterman, Astrid van Hylckama Vlieg, Suzanne C. Cannegieter, Frits R. Rosendaal

**Affiliations:** 1 Department of Clinical Epidemiology, Leiden University Medical Center, Leiden, The Netherlands; 2 Department of Thrombosis and Haemostasis, Leiden University Medical Center, Leiden, The Netherlands; University College London, United Kingdom

## Abstract

**Background:**

Venous thrombosis is a common disease with a high mortality rate shortly after the event. However, details on long-term mortality in these patients are lacking. The aim of this study was to determine long-term mortality in a large cohort of patients with venous thrombosis.

**Methods and Findings:**

4,947 patients from the Multiple Environmental and Genetic Assessment study of risk factors for venous thrombosis (MEGA study) with a first nonfatal venous thrombosis or pulmonary embolism and 6,154 control individuals without venous thrombosis, aged 18 to 70 years, were followed up for 8 years. Death and causes of death were retrieved from the Dutch death registration. Standardized mortality ratios (SMRs) were calculated for patients compared with control individuals. Several subgroups were studied as well.

736 participants (601 patients and 135 controls) died over a follow-up of 54,948 person-years. The overall mortality rate was 22.7 per 1,000 person-years (95% CI 21.0–24.6) for patients and 4.7 per 1,000 person-years (95% CI 4.0–5.6) for controls. Patients with venous thrombosis had a 4.0-fold (95% CI 3.7–4.3) increased risk of death compared with controls. The risk remained increased up to 8 years after the thrombotic event, even when no additional comorbidities were present. The highest risk of death was found for patients with additional malignancies (SMR 5.5, 95% CI 5.0–6.1). Main causes of death were diseases of the circulatory system, venous thrombosis, and malignancies. Main limitation was a maximum age of 70 at time of inclusion for the first event. Therefore results can not be generalized to those in the highest age categories.

**Conclusions:**

Patients who experienced a first venous thrombosis had an increased risk of death which lasted up to 8 years after the event, even when no comorbidities were present at time of thrombosis. Future long-term clinical follow-up could be beneficial in these patients.

*Please see later in the article for the Editors' Summary*

## Abstract 2

Linda Flinterman and colleagues report on the long-term mortality rate for individuals who have experienced a first venous thrombosis or pulmonary embolism. They describe an ongoing elevated risk of death for individuals who had experienced a venous thrombosis or pulmonary embolism as compared to controls, for up to eight years after the event.

## Editors' Summary

**Background:**

The term venous thrombosis describes the clinical situation—more common during pregnancy, after surgery, or serious illness—in which a blood clot lodges in a vein. One specific type, which is more serious, involves the clot forming in a major vein in the lower leg and thigh and is termed a deep venous thrombosis. The clot can block blood flow and cause swelling and pain, but more seriously, can break off and move through the bloodstream, causing an embolism. An embolism can get stuck in the brain (and cause a stroke), lungs (and cause a pulmonary embolism), heart (to cause a heart attack), and/or other areas of the body, leading to severe damage.

Venous thrombosis is known to be associated with considerable short-term morbidity and mortality: the mortality rate after venous thrombosis is about 20% within one year and studies to date have suggested that the mortality rate is two to four times higher for patients with pulmonary embolism, of whom 10%–20% die within three months after the event. Many factors are associated with venous thrombosis, and the underlying cause of the thrombosis affects survival; for example, those with thrombotic events provoked by surgery or trauma have a lower mortality risk than patients with thrombosis caused by malignancy. Furthermore, about 10%–20% of patients who have had a venous thrombosis develop a recurrence within five years and up to 50% develop post-thrombotic syndrome—long-term swelling, pain, and changes in skin color.

**Why Was This Study Done?:**

It is currently unknown whether the poor prognosis associated with venous thrombosis is limited to the months following the thrombotic event, or persists for years afterwards. So in this study, the researchers sought to answer this question by analyzing the long-term survival in a large cohort of patients who had experienced a first venous thrombosis and who were all followed for up to eight years.

**What Did the Researchers Do and Find?:**

The researchers used the Multiple Environmental and Genetic Assessment of risk factors for venous thrombosis study (MEGA study), which was a case-control study involving 4,965 consecutive patients aged 18 to 70 years who were objectively diagnosed with a deep venous thrombosis or pulmonary embolism and recruited from six anticoagulation clinics in the Netherlands between March 1999 and September 2004. The control group consisted of partners of patients (*n* = 3,297) and a random control group matched on age and sex (*n* = 3,000). The researchers obtained causes of death from the Central Bureau of Statistics and for the observation period (30 days after the venous thrombosis, to either death or end of follow-up between February 2007 and May 2009) compared cause-specific death rates of the patients to those of the general Dutch population. The researchers devised specialist survival models (called Kaplan-Meier life-tables) and calculated standardized mortality ratios (SMRs—the ratio of the observed number of deaths over the number of deaths expected) to estimate relative rates of all cause mortality by type of the initial thrombosis and the underlying cause.

Using these methods, the researchers found that the overall mortality rate in patients with thrombosis was substantially greater than in the control group (22.7 per 1,000 person-years compared to 4.7 per 1,000 person-years). Apart from malignancies, the researchers found that the main causes of death were diseases of the circulatory and respiratory system. Patients with venous thrombosis and malignancy had the highest risk of mortality: 55% died during follow-up. Patients with venous thrombosis without malignancy had an overall 2-fold increased risk of mortality compared to the control group and this risk was comparable for patients with different forms of thrombosis (such as deep venous thrombosis and pulmonary embolus). According to the researchers' calculations, the relative risk of death was highest during the first three years after thrombosis, but for those with thrombosis of unknown cause, the risk of death increased by two-fold up to eight years after the thrombosis. Furthermore, the researchers found that the highly increased risk of death for those with pulmonary embolism is mainly only for the first month as long-term survival is similar to that of patients with a deep venous thrombosis.

**What Do These Findings Mean?:**

These findings show that patients who have experienced a venous thrombosis for the first time have an increased risk of death, which may last up to eight years after the event. These findings have important clinical implications and suggest that long-term clinical follow-up could be beneficial in patients who have experienced a venous thrombosis for the first time.

**Additional Information:**

Please access these Web sites via the online version of this summary at http://dx.doi.org/10.1371/journal.pmed.1001155.

- Wikipedia provides information about venous thrombosis note that Wikipedia is a free online encyclopedia that anyone can edit; available in several languages)
- Medline has patient-friendly information about deep venous thrombosis and pulmonary embolism

## Introduction

Venous thrombosis is a multicausal disease that occurs in one to three per 1,000 persons per year [1]–[3]. Venous thrombosis is associated with considerable morbidity and mortality. About 10%–20% of patients develop a recurrence within 5 y [4]–[6], and up to 50% develop post-thrombotic syndrome within several months after the thrombotic event [7]. The mortality rate after venous thrombosis is about 20% within 1 y [2],[8]. Mortality is 2- to 4-fold higher for patients with pulmonary embolism (PE), of whom 10%–20% die within 3 mo after the event, than for patients with a deep vein thrombosis (DVT) of the leg [2],[9]–[11]. Malignancy is the main cause of death; however, when only patients without malignancy are followed, 12% die within a year after the thrombosis [2],[12]. Another predictor is the underlying cause of the first thrombosis, where those individuals with thrombotic events provoked by surgery or trauma have a lower 3-y mortality risk than those with idiopathic thrombosis [2]. These figures imply that venous thrombosis has a major impact on survival. It is currently unknown whether this poor prognosis is limited to the period shortly after the thrombotic event, or persists for extended periods.

In the present study we determined long-term survival in a large cohort of consecutive patients with a first venous thrombosis compared with age- and sex-matched individuals without venous thrombosis, who were all followed for up to 8 y.

## Methods

### Ethics Statement

This study was approved by the Ethics Committee of the Leiden University Medical Center and written informed consent was obtained from all the participants. The investigation has been conducted according to the principles expressed in the Declaration of Helsinki.

### Study Population and Data Collection

We used a cohort consisting of all patients and control individuals from the Multiple Environmental and Genetic Assessment of risk factors for venous thrombosis study (MEGA study). Details of the study are described elsewhere [12],[13]. In short, the MEGA study was set up as a case-control study including 4,965 consecutive patients aged 18 to 70 y with a first objectively verified venous thrombosis of the leg or PE and 6,297 control individuals recruited between March 1999 and September 2004.

Patients were recruited from six anticoagulation clinics in the Netherlands, which exclusively monitor out-patient treatment with vitamin K antagonists in well-defined geographical regions. Patients were included in the study after a median period of 1 mo (range 13–64 d) after the diagnosis of thrombosis.

The control group consisted of partners of patients (*n* = 3,297) and a random control group matched on age and sex (*n* = 3,000), recruited between January 2002 and December 2004 using random digit dialing. All patients and controls filled in a detailed questionnaire on risk factors for venous thrombosis and several comorbidities present at time of venous thrombosis (patients) or at time of inclusion in the study (controls).

Vital status was ascertained through community registries, where all inhabitants are registered. Causes of death were obtained from the Central Bureau of Statistics Netherlands, the national repository for death certificates. Both primary and secondary causes of deaths were retrieved. Causes of death were coded according to the ICD-10 classification [14],[15].

In the current study an additional control group was used. We compared cause-specific death rates of the patients to those of the general Dutch population, which, because of the size of the reference group, allowed analyses of cause-specific death rates, for which the control group of the MEGA study was too small.

### Follow-Up and Statistical Analysis

The observation time was from 30 d after the venous thrombosis, or a similar date in the thrombosis-free cohort, to either death, end of follow-up (between February 2007 and May 2009), emigration (*n* = 164, 1.5%), or loss to follow-up (*n* = 173, 1.5%), whichever occurred first.

For 152 individuals (1.4%, nine patients) follow-up was less than 30 d, and they were excluded. Censoring due to emigration concerned 164 individuals (1.5%) and to loss to follow-up 173 individuals (1.5%). This figure implies that follow-up was complete for 97% of the cohort. Vital status was obtained from the community registries and date of retrieving vital status was used as end date of follow-up, if patients were still alive. It was not possible to retrieve all vital statuses at the same date. Therefore, the end date of follow-up lies between February 2007 and May 2009.

The cohort of thrombosis-free individuals has a mortality that was exactly equal to the general population (standardized mortality ratio [SMR] 1.0, 95% CI 0.9–1.2), and there were no differences in mortality within the thrombosis-free cohort, between those who were recruited as partners of thrombosis patients or by random digit dialing.

Cumulative incidences and mortality rates were calculated at 1, 5, and 8 y of follow-up. Survival was estimated and visualized by Kaplan-Meier life-tables and survival curves.

SMRs were calculated to estimate relative rates of all cause mortality, e.g., by type of initial thrombosis. The SMR is the ratio of the observed number of deaths over the number of deaths expected when the mortality rate in the cohort of patients, with its specific age and sex distribution, was the same as that in the reference group. SMRs were calculated for the complete cohort of patients and for several subgroups of venous thrombosis patients: (1) for patients with active malignancy at time of inclusion or diagnosed within 6 mo after thrombosis; (2) for patients with a provoked first thrombosis without malignancy; (3) for patients with an idiopathic first thrombosis; and (4) according to type of first venous thrombosis (PE or DVT). An idiopathic thrombosis was defined as a thrombosis not related to surgery, hospital admission, injury, plaster cast, active malignancy, oral contraceptive use, or pregnancy, all in the year previous to the thrombotic event or during puerperium. SMRs for these categories of patients were calculated with the mortality rates of the complete control group as a reference; and also using only the rates of the controls belonging to the same category (e.g., patients with a provoked event were compared to controls who also had had surgery or plaster cast, etc.). This latter analysis was performed to estimate the effect of venous thrombosis on survival conditional on other risk factors for thrombosis.

Hazard ratios (HRs) of death from all causes per year of follow-up were calculated to estimate the decrease of mortality over time for the different subgroups. To calculate the HRs per year the hazard of dying in year X was calculated with all persons that survived up to year X. If they survived the whole of year X they were censored at the end of that year.

To calculate the reduction in median life expectancy we used the average life table of the birth cohorts of 1935–1965 of the Dutch population to create a population comparable in life expectancy to the MEGA study. To estimate the median life expectancy for the nonmalignant patients we multiplied the death rate per year for the Dutch cohort from the mean age at time of thrombosis by sex. The median life expectancy is the age at which half of a birth cohort of newborns had died. For this calculation we assumed an equal distribution of relative mortality after thrombosis in our population.

The influence of the presence of concurrent disease at the time of thrombosis on mortality was assessed by contrasting the patient cohort with the thrombosis-free cohort by Cox regression in strata of participants with or without concurrent diseases present. In addition, the annual HRs were adjusted for the number of concurrent diseases present at time of thrombosis in the Cox model. Concurrent diseases were diabetes, liver disease, kidney disease, heart failure, rheumatoid arthritis, chronic bronchitis, emphysema, myocardial infarction, stroke or hemorrhage of the brain, surgery 3 mo prior to thrombosis, and multiple sclerosis.

HRs were adjusted for sex and age. The assumption of proportionality was tested both visually from the Kaplan-Meier curve, and statistically, with the proportional hazard test provided by the software package used.

For the analysis of cause-specific mortality SMRs were calculated with the rates from the general population as reference. Analyses were performed using STATA 10.1. (Stata Corporation).

## Results

### Baseline Characteristics

4,947 patients with a first venous thrombosis and 6,154 controls were followed during a total period of 26,515 and 28,433 person-years, respectively. The baseline characteristics of patients and controls are described in Table 1. Median duration of follow-up was 5.5 y (range 1 mo–9.9 y) for patients and 4.4 y (range 1 mo–9.1 y) for controls. 601 patients (12%) and 135 controls (2%) died. Median time between inclusion and death was 1.7 y (range 36 d–9.2 y) for patients and 2.9 y (range 57 d–8.1 y) for controls.

**Table 1:** Baseline characteristics of the study population.

Characteristics	Percent Patients, *n* = 4,947 (*n*)	Percent Controls, *n* = 6,154 (*n*)
Sex (% men)	46 (2,271)	46 (2,854)
Mean age at index (range)^a^	49 (18–70)	47 (18–70)
Median age at index (range)^a^	50 (18–70)	48 (18–70)
Malignancy^b^	13 (619)	4 (233)
Diabetes	3.7 (183)	3.2 (195)
Liver disease	0.5 (27)	0.3 (20)
Kidney disease	1.2 (59)	0.4 (23)
Myocardial infarction	2.8 (137)	1.8 (113)
Heart failure	1.2 (76)	1.0 (60)
Stroke or hemorrhage of the brain	3.0 (154)	1.8 (115)
Chronic bronchitis	5.1 (253)	2.7 (165)
Emphysema	1.3 (66)	0.6 (35)
Multiple sclerosis	0.5 (30)	0.3 (17)
Rheumatoid arthritis	3.0 (144)	2.1 (132)

a Index date was date of thrombosis for patients and date of participation in the study for control individuals.

b Malignancy at time of or within 6 mo after the index date types of cancer have been previously reported [12].

### Overall Mortality

**Figure 1:**
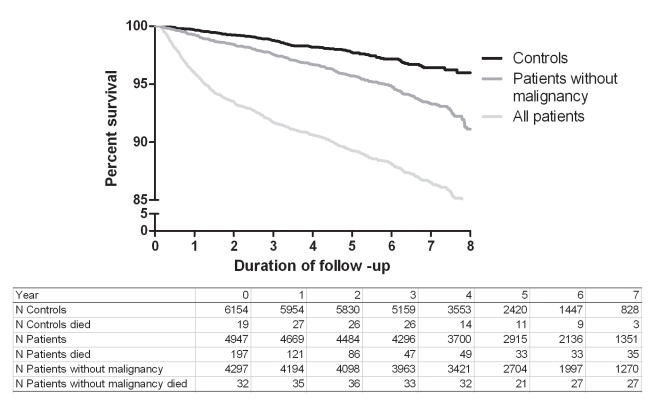
Kaplan-Meier survival curves for patients and controls.

The overall mortality rate after thrombosis was 22.7 per 1,000 person-years (95% CI 21.0–24.6), and the overall mortality rate for the control individuals was 4.7 per 1,000 person-years (95% CI 4.0–5.6). Figure 1 shows an increased risk of mortality for all patients compared with controls and for patients without malignancy compared with control individuals up to 8 y after the thrombotic event.

### Mortality of Patients with Malignancy

Patients with venous thrombosis and malignancy had, as expected, the highest risk of mortality. Overall, 55% of the patients' with malignancy and thrombosis died during follow-up, half of whom during the first year after thrombosis (Table 2). Patients with malignancy had a 17-fold increased risk of death (SMR 17.2; 95% CI 15.5–19.1) compared to the control group. Remarkably, when patients with malignancy and thrombosis were compared with individuals with malignancy without thrombosis they still had a five times higher rate of death (SMR 5.5; 95% CI 5.0–6.1) (Table 3).

**Table 2:** Cumulative incidences of mortality for different subgroups and controls overall, during the first year and during the first 5 y of follow-up.

Groups	*n* at Risk	1 y		5 y		Overall (8 y)	
		*n* Deaths	Percent Cumulative Incidence (95% CI)	*n* Deaths	Percent Cumulative Incidence (95% CI)	*n* Deaths	Percent Cumulative Incidence (95% CI)
Patients	4,947	197	4.0 (3.4–4.5)	500	10.1 (9.3–10.9)	601	12.1 (11.6–13.4)
Malignancy+	650	165	25.4 (22.0–28.7)	332	51.1 (47.2–54.9)	358	55.1 (51.3–58.9)
Malignancy−	4,297	32	0.7 (0.5–1.0)	168	3.9 (3.3–4.5)	243	5.7 (5.0–6.3)
Provoked^a^	2,949	21	0.7 (0.4–1.0)	94	3.2 (2.6–3.8)	122	4.1 (3.4–4.9)
Idiopathic^b^	1,348	11	0.8 (0.3–1.3)	74	5.5 (4.3–6.7)	121	9.0 (7.5–10.5)
Controls	6,154	19	0.3 (0.2–0.4)	112	1.8 (1.5–2.2)	135	2.2 (1.8–2.6)

a Individuals with malignancy excluded.

b Idiopathic venous thrombosis were those without malignancy, surgery, hospital admission, injuries, plaster, oral contraceptive use, and pregnancy or puerperium.

**Table 3:** Standardized mortality ratios.

Groups	*n* Patients (Events)	SMR Overall (95% CI)^a^	SMR Specific (95% CI)^b^
Overall	4,947 (601)	4.0 (3.7–4.3)	4.0 (3.7–4.3)
Malignancy+	650 (358)	17.2 (15.5–19.1)	5.5 (5.0–6.1)
Malignancy−	4,297 (243)	1.9 (1.7–2.1)	2.2 (1.9–2.5)
DVT	2,505 (144)	1.9 (1.7–2.3)	2.3 (1.9–2.7)
PE	1,257 (72)	1.9 (1.5–2.4)	2.2 (1.7–2.8)
DVT+PE	535 (27)	1.6 (1.1–2.3)	1.8 (1.2–2.6)
Provoked^c^	2,949 (122)	1.9 (1.6–2.2)	2.1 (1.8–2.5)
Idiopathic^d^	1,348 (121)	1.9 (1.6–2.3)	2.2 (1.8–2.6)

a SMRs are calculated with the nonthrombosis cohort as a reference.

b SMRs are calculated with the controls with the same selection criteria as the patients as a reference.

c Individuals with malignancy excluded.

d Idiopathic venous thrombosis were those without malignancy, surgery, hospital admission, injuries, plaster, oral contraceptive use, and pregnancy or puerperium.

DVT, patients with a first venous thrombosis of the leg without malignancies; DVT+PE, patients diagnosed with both PE and DVT without malignancies; PE, patients with a first PE without a diagnosis of a DVT without malignancies.

### Mortality Rates for Patients without Malignancy

Patients with venous thrombosis without malignancy had an overall 2-fold increased risk of mortality compared to the control group (Table 3). The risk was comparable for patients with different forms of thrombosis (DVT versus PE) and for patients with a provoked or an idiopathic thrombosis.

Table 4 shows the HRs year by year during follow-up. The relative risk of death was highest during the first 3 y, in all groups. Overall, patients with thrombosis had a persistent elevation in the risk of death, except for those with transient provoking factors; in this group the risk became, over time, similar to that of individuals who had provoking factors at baseline but did not suffer thrombosis. In contrast, for those who suffered idiopathic thrombosis, the risk of death remained over 2-fold increased up to 8 y after the thrombosis.

**Table 4:** Hazard ratios calculated per year with the control group as a reference.

Group	Controls	Year of Follow-up								Overall
		1	2	3	4	5	6	7	8	
		HR^a^ (95% CI)	HR (95% CI)	HR (95% CI)	HR (95% CI)	HR (95% CI)	HR (95% CI)	HR (95% CI)	HR (95% CI)	
Overall	All^b^	14.4 (7.1–29.2)	7.1 (3.7–13.6)	4.2 (2.3–7.5)	1.7 (1.0–3.1)	2.5 (1.4–4.8)	1.8 (0.9–3.6)	2.1 (1.0–4.6)	3.8 (0.5–30.8)	4.3 (3.4–5.5)
Malignancy	All	116.6 (56.5–240.9)	45.6 (22.9–90.7)	28.5 (14.8–54.9)	3.5 (1.6–7.9)	11.7 (5.1–27.1)	5.2 (2.0–13.6)	2.4 (0.6–9.0)	2.6 (0.1–77.7)	26.0 (20.1–33.7)
Malignancy	Malignancy^c^	18.4 (4.6–74.4)	11.3 (2.8–45.9)	5.5 (1.7–18.9)	2.2 (0.5–9.9)	2.2 (0.5–9.8)	—d	0.4 (0.1–1.7)	—d	6.6 (3.9–11.3)
Provoked^e^	All	3.7 (1.6–8.3)	3.2 (1.5–6.9)	1.8 (0.9–3.7)	1.2 (0.6–2.4)	2.7 (1.3–5.4)	1.0 (0.4–2.6)	1.8 (0.7–4.8)	3.2 (0.3–34.8)	2.1 (1.7–2.9)
Provoked^e^	Risk factors	5.7 (1.3–24.6)	3.3 (1.0–11.3)	1.6 (0.6–4.4)	1.4 (0.4–4.7)	1.7 (0.6–4.4)	1.0 (0.3–4.0)	—d	0.3 (0.0–4.1)	2.3 (1.5–3.6)
Idiopathic^f^	All	2.9 (1.1–7.9)	2.4 (1.0–5.7)	2.5 (1.2–5.4)	1.7 (0.8–3.6)	1.2 (0.5–2.9)	1.7 (0.7–3.8)	3.4 (1.4–8.2)	3.6 (0.4–31.7)	2.2 (1.6–3.0)
Idiopathic^f^	No risk factors	3.1 (1.0–10.2)	2.5 (0.9–7.1)	4.1 (1.5–11.3)	1.5 (0.7–3.3)	1.9 (0.6–5.8)	1.7 (0.7–4.0)	2.9 (1.1–7.6)	—d	2.5 (1.8–3.6)

a All HRs were adjusted for age and sex and number of comorbidities.

b All, all controls were taken into account.

c Selected, controls with the same characteristics as the patient subgroup were taken into account, e.g., selected malignancy are only controls with malignant disease.

d HR could not be calculated, because there were no events.

e Individuals with malignancy excluded.

f Idiopathic venous thrombosis were those without malignancy, surgery, hospital admission, injuries, plaster, oral contraceptive use and pregnancy and were not during puerperium.

The reduction of median life expectancy for those without malignancy was 5 y for men and women. The estimated median life expectancy was 76 for men and 79 for women compared to 81 and 84 y respectively for the Dutch population.

### Comorbidity

Patients with thrombosis and without malignancy have an increased risk of death, which could be explained by concurrent other major disorders (Table 4). When we stratified our study population for those with and without concurrent disorders we found no difference in risk of death in the stratum of participants with comorbidities (HR 1.2 [0.9–1.7] for patients with venous thrombosis compared to controls). However, for patients without concurrent major disorders at time of thrombosis overall a HR of 2.5 (95 CI% 1.9–3.4) was found compared to controls without concurrent disorders and thrombosis. The increased risk of death among patients with venous thrombosis can therefore not be fully explained by the presence of these concurrent disorders.

### Causes of Death

The main primary cause of death was malignancy (*n* = 392, 65%) followed by diseases of the circulatory (*n* = 80, 13%) and respiratory (*n* = 34, 6%) systems (Table 5). 24 patients died of PE (which is classified under circulatory) either as primary cause (*n* = 7) or as a complication (*n* = 17).

**Table 5:** Increased mortality per cause of death compared with the Dutch population.

Cause of Death (Primary)	*n* (%) Overall	SMR Overall (95% CI)	*n* (%) Patients without Malignancy	SMR Patients without Malignancy (95% CI)
I00–I99 Diseases of the circulatory system	80 (13)	2.0 (1.6–2.5)	72 (30)	2.2 (1.7–2.7)
I21 Acute myocardial infarction	26 (4)	2.4 (1.6–3.5)	23 (10)	2.5 (1.7–3.8)
I61 Cerebral Hemorrhage	4 (0.6)	1.9 (0.7–5.0)	2 (1)	1.1 (0.3–4.5)
I63 Cerebral infarction	1 (0.1)	0.7 (0.1–5.0)	1 (0.5)	0.8 (0.1–5.9)
I64 Stroke	2 (0.3)	0.8 (0.2–3.3)	2 (1)	1.0 (0.2–4.0)
I26 and I80 Venous thromboembolism	7 (1)	5.5 (2.6–11.4)	7 (3)	6.5 (3.1–13.7)
C00–C99 Neoplasms	392 (65)	5.5 (5.0–6.0)	69 (29)	1.1 (0.9–1.4)
J00–J99 Diseases of the respiratory system	34 (6)	3.3 (2.3–4.6)	29 (12)	3.3 (2.3–4.8)
J44 Chronic obstructive pulmonary disease	19 (3)	3.7 (2.4–5.8)	16 (7)	3.7 (2.3–6.1)
J18 Pneumonia	5 (0.8)	2.1 (0.9–4.9)	5 (2)	2.4 (1.0–5.9)

Cause-specific mortality was compared with data from the general population. Patients had two times higher rates of deaths from diseases of the circulatory system (*n* = 80, SMR 2.0, 95% CI 1.6–2.5) and three times higher death rates of diseases from the respiratory system (*n* = 34, SMR 3.3, 95% CI 2.3–4.6) than the general population. Venous thrombosis and malignancy were the causes of death with the highest SMR compared to the general population (Table 5). Patients who died of diseases of the respiratory system mainly died of chronic obstructive pulmonary diseases or pneumonia as primary cause of death. Five patients died of either a subarachnoid or intracerebral hemorrhage of whom three were on anticoagulation treatment at time of death.

For patients without malignancy the main causes of death were diseases of the respiratory system and diseases of the circulatory system (SMR 3.3, 95% CI 2.3–4.8 and 2.2, 95% CI 1.7–2.7, respectively). Compared with the general population they did not have an increased risk of death due to malignancies (Table 5).

## Discussion

We studied long-term mortality after a first venous thrombosis in 4,947 patients followed for a median period of 5.5 y, compared with a thrombosis-free cohort of 6,154 individuals. The overall mortality rates were 22.7 per 1,000 person-years for all patients. Overall, the death rate in patients was 4.0-fold increased and in those without a malignancy over two-fold increased. In all patients except those with a transient provoking risk factor underlying the initial event, death rates remained elevated up to 8 y after the thrombotic event.

Few studies have studied the long-term risk of mortality after venous thrombosis. In a previous study from Norway, the cumulative incidences at 1 y were much higher than those we found. In the Norwegian study a cumulative incidence at 1 y of 14.5% was found for cases with an idiopathic venous thrombosis, of 9.7% for provoked cases, and of 63.4% for cancer patients, while we found cumulative incidences of 0.8%, 0.7%, and 25.4%, respectively [2]. These differences result from the inclusion of inpatients in the Norwegian study, thereby also counting early deaths, and the inclusion of patients of all ages, while our study was restricted to patients younger than 70 y at the time of the first event. This study design implies that overall mortality in patients with venous thrombosis is even higher than we report. Because of our extended follow-up for up to 8 y after the thrombotic event, our most important observation is that increased mortality for thrombosis patients persists for a prolonged time. Furthermore, we showed that, when only the long-term survival is taken into account, there is no longer a difference in survival for patients with a DVT and PE. This finding indicates that the highly increased risk of death for those with PE is mainly present during the first month after venous thrombosis. Recently, an Austrian study did not find an increased risk of long-term mortality for patients with venous thrombosis [16]. However, they included patients at a median time of 14 mo after thrombosis up to 6 y after the thrombotic event. Because of this delayed inclusion only long-term survivors of thrombosis were included in the Austrian study and therefore no increase in mortality was found.

We confirmed previous observations that patients with malignancy and venous thrombosis have a very poor prognosis, substantially worse than patients with cancer without thrombosis, with a 5.5-fold difference in our study [17],[18]. Although death from recurrent thrombosis was clearly elevated after a first thrombotic event, most patients died of other causes, mainly of the circulatory system. While one is tempted to explain this by preexisting comorbid conditions, death rates were also persistently elevated after idiopathic thrombosis and in those without any major illnesses.

Main causes of death, apart from malignancies, were diseases of the circulatory and respiratory system. These results are in line with previous studies that described associations between risk factors for venous and arterial thrombosis as well as an increased risk of arterial thrombosis for those who experienced venous thrombosis [19]–[21]. Alternatively, misclassification of cause of death may explain (part of) the results, especially when no further research into the cause of death was performed, although one would expect this to affect the results in the opposite direction (patients with previous PE may be more readily misclassified as having died of PE than of other lung diseases than those without a history of PE).

Our study may have suffered from some limitations: as discussed above causes of death were not objectively verified. However misclassification by the physician determining the cause of death is most likely to have been equal in this population and in the general population, which would have led to an underestimation of the risks we have found. Furthermore, we only recruited patients after discharge from hospital, and therefore overall mortality is underestimated. Moreover, our results cannot be extrapolated to patients older than 70 y at the time of thrombosis, or to children.

Among the major strengths of this study are the large size of the cohort, and the long follow-up period. Mortality data were retrieved from the national registry where 98.5% of participants were found. Therefore, loss to follow-up was minimal and dates of death were accurate. To our knowledge, this has been the first study to calculate mortality rates compared with the general population and compared to specific control groups. Therefore, we were able to define overall risks of death up to 8 y after thrombosis as well as the risk for several subgroups. Our results underline the major consequences of venous thrombosis, not only with regard to morbidity but also to mortality.

## Abbreviations

DVT: deep vein thrombosis
HR: hazard ratio
MEGA: Multiple Environmental and Genetic Assessment
PE: pulmonary embolism
SMR: standardized mortality ratio
